# Relationship of Oxidative Stress, Inflammation, and the Risk of Metabolic Syndrome in Patients with Oral Cancer

**DOI:** 10.1155/2018/9303094

**Published:** 2018-05-21

**Authors:** Bor-Jen Lee, Man-Yee Chan, Han-Yu Hsiao, Chia-Hua Chang, Li-Ping Hsu, Ping-Ting Lin

**Affiliations:** ^1^Department of Critical Care Medicine, Taichung Veterans General Hospital, Taichung 40705, Taiwan; ^2^School of Medicine, Chung Shan Medical University, Taichung 40201, Taiwan; ^3^Division of Oral and Maxillofacial Surgery, Department of Dentistry, Taichung Veterans General Hospital, Taichung 40705, Taiwan; ^4^School of Dentistry, College of Oral Medicine, Chung Shan Medical University, Taichung 40201, Taiwan; ^5^Department of Nutrition, Chung Shan Medical University, Taichung 40201, Taiwan; ^6^Department of Nutrition, Chung Shan Medical University Hospital, Taichung 40201, Taiwan

## Abstract

Oral cancer is the fifth leading cause of cancer death in Taiwan, and the prevalence of metabolic syndrome (MS) has also increased globally. The purpose of this study was to investigate the correlations between the components of MS and oxidative stress and inflammation in patients with oral cancer based on their areca-nut-chewing habits. Two hundred patients diagnosed with oral cancer were recruited, and metabolic parameters, oxidative stress, antioxidant enzyme activities, and inflammatory markers were measured. 63% of the subjects have concomitant MS. Subjects who had an areca-nut-chewing habit had significantly higher levels of fasting glucose (*p* = 0.04), oxidative stress (*p* = 0.02), and inflammatory markers (*p* = 0.02) than those who never chewed. High-density lipoprotein-cholesterol level (*p* = 0.03) and superoxidase dismutase activity (*p* = 0.02) were significantly lower in individuals who had chewed or were currently chewers. Areca-nut-chewing habit was associated with the increased risks for MS and hypertriglyceridemia; the components of MS were positively correlated with oxidative stress and inflammation. In conclusion, patients with oral cancer who had an areca-nut-chewing habit exhibited higher levels of oxidative stress and inflammation, which might be related to an increased risk of MS.

## 1. Introduction

According to the latest cancer statistics from the American Cancer Society, Americans accounted for the estimated 30% of the new cases of oral cancer and 2% of the oral-cancer-related deaths that occurred in the US in 2017 [1]. By contrast, almost 60% of all new cases of oral cancer occurred in Asia, and 68% of oral-cancer-related deaths occurred in the Asian population [2]. In Taiwan, oral cancer is the fifth leading cause of cancer deaths in both sexes and is the fourth leading cause of cancer deaths in men [3]. The areca-nut-chewing habit is an independent risk factor for oral cancer [4] as the constituents of areca nut ingredients, such as areca alkaloids, have been shown to be carcinogenic to humans; furthermore, areca-nut-specific nitrosamines are genotoxic and induce tumor progression [5]. In addition to the carcinogenic effects of areca nut chewing, harmful relationships between this habit and metabolic syndrome (MS) [6-9], type 2 diabetes [9, 10], and cardiovascular disease [9, 11, 12] have also been observed. In clinical practice, we usually recommend that patients with oral cancer should abstain from areca nut chewing. However, no research has followed the status of metabolic parameters of oral cancer patients after quitting areca nut chewing.

It is well known that tumor promotion is associated with prooxidant status [13], as reactive oxygen species (ROS) participate in the progression of carcinogenesis [13, 14]. During areca nut chewing, ROS are produced, which attack salivary proteins, change the structure of oral mucosa, and activate the inflammatory responses [15]. There is limited clinical research assessing the status of oxidative stress and inflammatory and metabolic parameters in individuals with oral cancer even if they quit areca nut chewing. Therefore, the aim of the present clinical study was to investigate the relationship between the areca-nut-chewing habit and oxidative stress, inflammation, and the risk of MS in the patients with oral cancer based on their areca-nut-chewing habits.

## 2. Materials and Methods

### 2.1. Participants

This was an observational study. Patients diagnosed with oral cancer and who underwent tumor resection were recruited from the Department of Dentistry at Taichung Veterans General Hospital. We excluded patients younger than 20 years of age or older than 80 years of age, as well as those with a history or current diagnosis of liver or renal disease and those taking antioxidant vitamins. Informed consent was obtained from each subject. This study was approved by the Institutional Review Board of Taichung Veterans General Hospital, Taiwan. The following data were recorded from the medical questionnaire administered to all subjects: age, blood pressure, areca-nut-chewing habits (including current users, those with a history of areca nut chewing (defined as quitting areca nut chewing ≥ 6 months prior), and nonusers), smoking and drinking habits, and exercise frequency. Body weight, height, and waist and hip circumferences were measured; the body mass index (BMI) and the ratio of waist to hip were calculated. The diagnostic criteria for MS in Taiwan are based on the guidelines of the Administration of Health Promotion, Ministry of Health and Welfare, Taiwan (2007). Subjects were considered to have MS if they had three of the following five characteristics: (1) abdominal obesity (waist circumference ≥ 90 cm in males and ≥80 cm in females), (2) impaired fasting glucose (fasting glucose (FG) ≥ 5.6 mM), (3) hypertriglyceridemia (triglyceride (TG) ≥ 1.7 mM), (4) low levels of high-density lipoprotein-cholesterol (HDL-C) < 1.0 mM in males and <1.3 mM in females, and (5) increased blood pressure (systolic blood pressure (SBP) ≥ 130 mmHg and diastolic blood pressure (DBP) ≥ 85 mmHg). Subjects taking antidiabetic, antihypertensive, and lipid-lowering medications were considered to have elevated FG, elevated blood pressure, and dyslipidemia, respectively.

### 2.2. Blood Collection and Biochemical Measurement

Fasting venous blood samples (15 mL) were collected in vacutainer tubes (Becton Dickinson, Rutherford, NJ, USA) with and without anticoagulant as required. Plasma and serum samples were prepared after centrifugation (3000 rpm, 4°C, and 15 minutes). Hematological parameters, such as FG and lipid profiles, were measured using an automated biochemical analyzer (Hitachi, Tokyo, Japan). Plasma MDA (malondialdehyde) was determined using the thiobarbituric acid reactive substance method [16]. The methods for measuring CAT, SOD, and GPx in red blood cells have been previously described [17–19]. First, red blood cells (RBC) were diluted with 25x sodium phosphate buffer for superoxide dismutase (SOD) and glutathione peroxidase (GPx) measurement and 250x sodium phosphate buffer for catalase (CAT) measurement. SOD activity was measured by the decreasing rate of pyrogallol autoxidation under alkali conditions (pH 8.2) and then analyzed spectrophotometrically at 325 nm [18]. CAT activity was measured spectrophotometrically at 240 nm with hydrogen peroxide as substrate [19]. GPx activity was measured with cumene hydroperoxide as substrate coupled with glutathione reductase and then analyzed spectrophotometrically at 340 nm [17]. Protein contents of RBC were determined based on the biuret reaction of the BCA Protein Assay Reagent Kit (Thermo Scientific, Rockford, IL, USA), and the antioxidant enzyme activities were expressed as the units/mg of protein. Serum level of high-sensitivity C-reactive protein (hs-CRP) was quantified by particle-enhanced immunonephelometry with an image analyzer (Dade Behring, Deerfield, IL, USA).

### 2.3. Statistical Analysis

Data were analyzed using SigmaPlot software (version 12.0, Systat Software, Inc., San Jose, CA, USA). The normality of the distribution of the variables was evaluated using the Shapiro-Wilk test. For categorical response variables, the differences between the two groups were assessed using the Chi-square test or Fisher's exact test. One-way ANOVA analyses were performed to examine the differences in the patients who currently, formerly, and never chewed areca nut, and post hoc tests were further used to compare the differences among the three groups. To examine the correlations between the components of MS and oxidative stress and inflammation, Spearman rank order correlation was used. Logistical regression analyses were further used to examine the correlation between the areca-nut-chewing habit and the components of MS after adjusting for age and gender. The results were considered statistically significant at *p* < 0.05. The values are presented in the text as the means ± standard deviation (medians).

## 3. Results

### 3.1. Demographic Characteristcs of the Subjects

Two hundred patients with oral cancer were included and were made to complete the data analyses in the present study. A total of 95% of the oral cancer patients were male with a mean age of 54 years old. A total of 63% of the subjects suffered from MS, 58% suffered from abdominal obesity, 76% suffered from borderline hypertension, 66% suffered from borderline hyperglycemia, 49% suffered from borderline hypertriglyceridemia, and 47% suffer from low levels of HDL-C. With regard to areca nut habits, 19% of the subjects were current users, 71% of the subjects were former users, and 11% of the subjects had never chewed areca nut. In Table 1, the demographic characteristics of subjects are stratified by their areca-nut-chewing habits. Subjects who had a current areca-nut-chewing habit had a significantly lower mean age and higher frequency of smoking and alcohol use. A higher incidence of abdominal obesity, elevated blood pressure, and increased recurrence rate for oral cancer and incidence of hypertensive disease history were observed in former areca nut chewers.

### 3.2. Levels of Metabolic Parameters

The levels of glucose and lipid profiles of the subjects as stratified by their areca-nut-chewing status are shown in Table 2. Subjects who were currently chewing or had formerly chewed areca nut had a significantly higher FG level than those who never chewed (*p* = 0.04). The level of HDL-C was significantly lower in subjects who were currently chewing areca nut than in those who had never chewed (*p* = 0.03).

### 3.3. Levels of Oxidative Stress, Inflammation, and Antioxidant Enzymes

**Figure 1:**
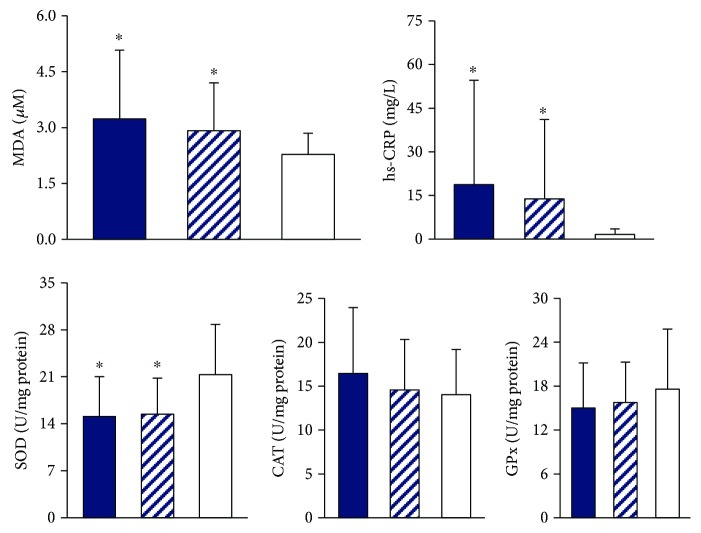
Levels for oxidative stress, inflammatory markers, and antioxidant enzyme activities based on areca nut use. CAT, catalase activity; MDA, malondialdehyde; GPx, glutathione peroxidase; hs-CRP, high-sensitivity C-reactive protein; SOD, superoxide dismutase. ^∗^Values were significantly different from subjects who never chewed areca nut. (Blue-colored bar) current chewers, (striped bar) former chewers, and (white bar) never chewers.

The levels of oxidative stress, inflammatory markers, and antioxidant enzyme activities are shown in Figure 1. Subjects who were currently chewing or had formerly chewed areca nut had significantly higher levels of oxidative stress (MDA, *p* = 0.02) and inflammation (hs-CRP, *p* = 0.02) than those who had never chewed; meanwhile, a significantly lower level of SOD activity was found in current and former chewers (*p* = 0.02).

### 3.4. Correlations between Components of Metabolic Syndrome and Oxidative Stress and Inflammation

The correlations between components of MS and both oxidative stress and inflammation are shown in Table 3. MS was significantly positively correlated with oxidative stress (*r* = 0.14, *p* = 0.01) and inflammatory markers (*r* = 0.17, *p* = 0.02). Regarding specific parameters of MS, hyperglycemia (*r* = 0.13, *p* = 0.01) and hypertriglyceridemia (*r* = 0.14, *p* = 0.01) were significantly positively correlated with oxidative stress. Furthermore, abdominal obesity (*r* = 0.12, *p* = 0.02), hyperglycemia (*r* = 0.24, *p* < 0.01), and low HDL-C (*r* = 0.26, *p* < 0.01) were significantly positively correlated with inflammation.

We also examined the correlation between areca nut use and the risk of MS after adjusting for age and gender and the data are shown in Table 4. Subjects with a current areca-nut-chewing habit had significantly increased risks of MS (odds ratio = 2.30, *p* = 0.03) and hyperglycemia (odds ratio = 2.71, *p* < 0.01).

## 4. Discussion

Some community-based health population surveys have reported that oral precancerous lesions were significantly correlated with the components of MS and were related to areca nut chewing [10, 20–22]. Areca nut is a major contributor of oral cancer [4], and nearly 90% of patients with oral cancer in the present study had a history of areca nut chewing. Interestingly, 70% of those patients quit areca nut chewing for a median of 6.3 years. However, higher levels of oxidative stress and inflammatory status were still detected in individuals who quit areca nut chewing (Figure 1). Approximately 60% of the patients in the present study suffer from MS. Among the components of MS, the incidence of high blood pressure (81%) and abdominal obesity (62%) was significantly higher in those patients who had ever chewed areca nut, even those who had quit. Additionally, areca nut use was significantly related to an increase in the risk of MS, particularly hyperglycemia (Table 4), and the components of MS, such as abdominal obesity, hyperglycemia, hypertriglyceridemia, or low HDL-C level was significantly positively correlated with oxidative stress and inflammation (Table 3). Thus, we proposed that the patients with oral cancer who have a history of chewing areca nut might present higher levels of oxidative stress and inflammation, further increasing the risk of MS. Some molecular mechanisms have been thoroughly investigated, and studies have shown that areca nut contents of hydroxychavicol and arecoline compounds which may induce ROS production, cause cell cycle aberration and cell irregular cellular differentiation [23, 24], induce platelet aggregation [25] and inflammation [26, 27], and are related to the malignant transformation precancerous oral lesions [28].

Among the components of MS, hyperglycemia is the most significant abnormal metabolic condition in patients with oral cancer who have or had an areca-nut-chewing habit. Approximately 62% of patients with oral cancer who have a current or had a former areca-nut-chewing habit suffered from hyperglycemia (Table 1). Areca nut and the associated nitrosamines might promote glucose intolerance due to central obesity [29–31]. Approximately half of our subjects (58%) had abdominal obesity, 76% of whom had a history of areca nut chewing. The mechanism by which areca nut promotes hyperglycemia might be related to the areca alkaloids, which may *stimulate* the appetite via *the* inhibition of the GABA receptors [30]; established *oral chewing habit* can also increase appetite, which contributes to the risk of obesity or insulin resistance [31, 32].

A dose-dependent relationship between areca nut use and triglyceride were reported in the Nutrition and Health Survey in Taiwan (1993–1996) [6]. Although we did not detect a significantly higher level of triglyceride in the patients with oral cancer, we noted that current and former areca nut chewers had an elevated triglyceride level, which was higher than normal values (1.7 mM). In addition, a significantly lower level of HDL-C was also found in current areca nut chewers (Table 2). One possible mechanism of areca nut affecting metabolic processes is that arecoline may inhibit the differentiation of adipose tissue, induce adenylyl-cyclase-dependent lipolysis, and interfere with the insulin signaling pathway associated with glucose uptake, all of which contribute to hyperlipidemia and insulin resistance [33]. Apart from this, areca nut also increases the lipid profile burden through increasing oxidative stress and inflammation (Table 4). Although we cannot clarify the causal relationship between oxidative stress, inflammation, and the risk for MS in this cross-sectional study, the harmful effects of the areca nut were obvious in the patients with oral cancer who were currently chewing or had formerly chewed areca nut.

Since higher levels of oxidative stress and inflammation are significantly correlated with an increased risk of MS [34], antioxidant administration for patients with oral cancer are worth considering as intervention strategies. Antioxidants can reduce oxidative damage and inflammation, delay cancer progression, and prevent cancer recurrence by scavenging free radicals [35]. Many studies have successfully used vitamins or phytochemicals as antioxidative adjuvants for head and neck squamous cell carcinoma [36, 37]. Antioxidative nutrients, which can also be obtained from a healthy diet that includes plenty of fresh vegetables and fruits, have been proposed to reduce the risk of head and neck cancer [38]. Recently, a national survey from the US (NHANES 2001–2006) reported that antioxidant levels were inversely associated with the risk of MS and other related parameters, such as insulin resistance, elevated CRP, or hyperuricemia [39], as well as dietary antioxidants, which were also related to a lower prevalence of MS [40]. Thus, further intervention studies should focus on antioxidant supplementation in patients with oral cancer and on monitoring their antioxidant status, particularly individuals with a history of areca nut chewing.

## 5. Conclusions

In conclusion, oral cancer patients with a history of areca nut chewing may present higher levels of oxidative stress and inflammation, even after quitting. As higher oxidative stress and inflammation status may be correlated with an increased risk of MS, we recommend that patients with oral cancer not only stop areca nut chewing but also consume an antioxidant-rich diet, which might help reduce oxidative stress and inflammation and thereby lower the risk of MS.

## Figures and Tables

**Table 1:** Demographic characteristics of the subjects according to areca nut use.

	Current chewers (*n* = 38)	Former chewers (*n* = 141)	Never chewers (*n* = 21)	*p* values
Males (*n*, %)	37 (97%)	137 (97%)	15 (71%)	<0.01
Age (y)	50 ± 9 (51.0)^1,a^	55 ± 10 (56.0)^b^	54 ± 12 (55.0)^a,b^	0.03
SBP (mmHg)	132 ± 16 (132.0)	137 ± 20 (135.0)	131 ± 16 (127.0)	0.24
DBP (mmHg)	85 ± 12 (83.5)	88 ± 15 (86.0)	85 ± 9 (84.0)	0.55
Waist (cm)	91 ± 10 (89.0)	92 ± 11 (93.0)	96 ± 10 (96.0)	0.13
BMI (kg/m^2^)	25 ± 4 (24.4)	26 ± 7 (25.8)	25 ± 5 (24.3)	0.75
MS (*n*, %)	24 (63%)	90 (64%)	11 (52%)	0.60
Abdominal obesity^2^ (*n*, %)	17 (45%)	86 (61%)	12 (57%)	0.08
High blood pressure^3^ (*n*, %)	25 (66%)	113 (80%)	14 (67%)	0.04
Hyperglycemia^4^ (*n*, %)	27 (71%)	96 (68%)	9 (43%)	0.26
Hypertriglyceridemia^5^ (*n*, %)	18 (47%)	69 (49%)	11 (52%)	0.83
Low HDL-C^6^ (*n*, %)	21 (55%)	62 (44%)	11 (52%)	0.22
*Smoker* ^7^				<0.01
Current (*n*, %)	25 (66%)	56 (40%)	7 (33%)	
Ever (*n*, %)	11 (29%)	66 (47%)	6 (29%)	
Never (*n*, %)	2 (5%)	19 (13%)	8 (38%)	
*Alcohol use* ^8^				<0.01
Current (*n*, %)	16 (42%)	55 (39%)	4 (19%)	
Ever (*n*, %)	12 (32%)	48 (34%)	2 (10%)	
Never (*n*, %)	10 (26%)	38 (27%)	15 (71%)	
Exercise^9^ (*n*, %)	8 (21%)	59 (42%)	8 (38%)	0.06
Cancer recurrence (*n*, %)	1 (3%)	57 (40%)	2 (10%)	<0.01
*TNM stages* ^10^ (*n*, %)				0.90
Stage 0	0	2 (1.4%)	0	
Stage I	8 (21.1%)	29 (20.6%)	6 (28.6%)	
Stage II	10 (26.3%)	48 (34.0%)	4 (19.0%)	
Stage III	7 (18.4%)	18 (12.8%)	5 (23.8%)	
Stage IVA	12 (31.6%)	34 (24.1%)	6 (28.6%)	
Stage IVB	1 (2.6%)	3 (2.1%)	0	
Stage IVC	0	1 (0.7%)	0	
*Disease history*				
Hypertension (*n*, %)	5 (13%)	38 (27%)	2 (10%)	0.06
Diabetes (*n*, %)	5 (13%)	25 (18%)	2 (10%)	0.55
*Family history*				
Oral cancer (*n*, %)	1 (3%)	5 (4%)	0	0.67
Hypertension (*n*, %)	9 (24%)	39 (28%)	8 (38%)	0.49
Diabetes (*n*, %)	4 (11%)	28 (20%)	3 (14%)	0.37

^1^Mean ± SD (median). ^2^Waist circumferences: ≥90 cm (males) and ≥80 cm (females). ^3^SBP ≥ 130 mmHg or DBP ≥ 85 mmHg, or taking antihypertensive drugs. ^4^Fasting glucose ≥ 5.6 mM or taking hypoglycemic drugs. ^5^TG ≥ 1.7 mM or taking antihyperlipidemia drugs. ^6^HDL-C: <1.0 mM (males) and <1.3 mM (females). ^7^Smoker: individuals regularly smoking one or 1 more cigarette per day. ^8^Alcohol use: individuals regularly consuming one or 1 more drink per day. ^9^Exercise: individuals exercise regularly at least 3 times per week. ^10^Six subjects who had formerly chewed areca nut had no TNM data. BMI, body mass index; DBP, diastolic blood pressure; SBP, systolic blood pressure; TNM, tumor-node-metastasis. ^a,b^Values with different superscripts were significantly different among the three groups.

**Table 2:** Levels of metabolic parameters based on areca nut use.

	Current chewers (*n* = 38)	Former chewers (*n* = 141)	Never chewers (*n* = 21)	*p* values
FG (mM)	6.6 ± 1.7 (5.9)^1,a^	7.3 ± 3.6 (6.0)^a^	5.6 ± 0.9 (5.4)^b^	0.04
TC (mM)	4.8 ± 0.9 (4.6)	4.7 ± 1.0 (4.7)	4.7 ± 0.9 (4.8)	0.89
TG (mM)	2.2 ± 1.3 (1.9)	2.4 ± 2.1 (1.7)	1.7 ± 1.0 (1.4)	0.37
LDL-C (mM)	3.0 ± 0.7 (2.8)	1.2 ± 0.9 (2.7)	2.9 ± 0.9 (2.8)	0.51
HDL-C (mM)	1.0 ± 0.2 (1.0)^a^	1.1 ± 0.3 (1.0)^a,b^	1.3 ± 0.3 (1.2)^b^	0.03
TC/HDL-C	5.2 ± 1.7 (5.0)	4.6 ± 1.5 (4.3)	4.3 ± 1.4 (3.9)	0.17

^1^Mean ± SD (median). FG, fasting glucose; HDL-C, high-density lipoprotein-cholesterol; LDL-C, low-density lipoprotein-cholesterol; TC, total cholesterol; TG, triglyceride. ^a,b^Values with different superscripts were significantly different among the three groups.

**Table 3:** Correlations between components of metabolic syndrome and oxidative stress and inflammation.

	Metabolic syndrome^2^	Abdominal obesity^3^	High blood pressure^4^	Hyperglycemia^5^	Hypertriglyceridemia^6^	Low HDL-C^7^
	*r* ^1^					
*Oxidative stress*						
MDA (*μ*M)	0.14^∗^	0.03	0.05	0.13^∗^	0.14^∗^	−0.04
*Antioxidant capacity*						
SOD (U/mg protein)	−0.09	−0.06	−0.02	−0.08	−0.04	−0.08
CAT (U/mg protein)	−0.03	−0.07	−0.03	−.001	0.01	0.02
GPx (U/mg protein)	0.05	0.01	−0.04	0.09	−0.01	0.05
*Inflammatory markers*						
hs-CRP (mg/L)	0.17^∗^	0.12^∗^	−0.03	0.24^∗∗^	−0.00	0.26^∗∗^

^1^Correlation coefficient (*r*). ^2^Yes = 1 and no = 0. ^3^Waist circumferences: ≥90 cm (males) and ≥80 cm (females). ^4^SBP ≥ 130 mmHg or DBP ≥ 85 mmHg, or taking antihypertensive drugs. ^5^Fasting glucose ≥ 5.6 mM or taking hypoglycemic drugs. ^6^TG ≥ 1.7 mM or taking antihyperlipidemia drugs. ^7^HDL-C: <1.0 mM (males) and <1.3 mM (females). CAT, catalase activity; MDA, malondialdehyde; GPx, glutathione peroxidase; hs-CRP, high-sensitivity C-reactive protein; SOD, superoxide dismutase. ^∗^*p* < 0.05 and ^∗∗^*p* < 0.01.

**Table 4:** Correlations between areca nut use and the risk of metabolic syndrome after adjusting for age and gender.

	Never use	Areca nut use		
		Odds ratio	95% CI	*p* values
Metabolic syndrome	1.00	2.30	0.12–3.27	0.03
Abdominal obesity^1^		1.21	0.58–2.54	0.61
High blood pressure^2^		1.66	0.76–3.65	0.21
Hyperglycemia^3^		2.71	1.29–5.69	<0.01
Hypertriglyceridemia^4^		0.83	0.39–1.78	0.63
Low HDL-C^5^		0.86	0.40–1.84	0.70

^1^Waist circumferences: ≥90 cm (males) and ≥80 cm (females). ^2^Systolic blood pressure ≥ 130 mmHg or diastolic blood pressure ≥ 85 mmHg, or taking antihypertensive drugs. ^3^Fasting glucose ≥ 5.6 mM or taking hypoglycemic drugs. ^4^Triglyceride ≥ 1.7 mM or taking antihyperlipidemia drugs. ^5^HDL-C: <1.0 mM (males) and <1.3 mM (females). CI, confidence interval; HDL-C, high-density lipoprotein-cholesterol.

## Data Availability

The datasets generated and/or analyzed during the current study are available from the corresponding author on reasonable request.
